# Overexpression of the *Aspergillus niger* GatA transporter leads to preferential use of D-galacturonic acid over D-xylose

**DOI:** 10.1186/s13568-014-0066-3

**Published:** 2014-08-23

**Authors:** Jasper Sloothaak, Mike Schilders, Peter J Schaap, Leo H de Graaff

**Affiliations:** 1 Microbial Systems & Synthetic Biology, Laboratory of Systems and Synthetic Biology, Wageningen University, Dreijenplein 10, Wageningen, NL-6703, HB, Netherlands

**Keywords:** D-galacturonic acid, Pectin, Sustainable resources, Aspergillus niger, Transmembrane transport

## Abstract

Pectin is a structural heteropolysaccharide of the primary cell walls of plants and as such is a significant fraction of agricultural waste residues that is currently insufficiently used. Its main component, D-galacturonic acid, is an attractive substrate for bioconversion. The complete metabolic pathway is present in the genome of *Aspergillus niger*, that is used in this study. The objective was to identify the D-galacturonic acid transporter in *A. niger* and to use this transporter to study D-galacturonic acid metabolism.

We have functionally characterized the gene An14g04280 that encodes the D-galacturonic acid transporter in *A. niger.* In a mixed sugar fermentation it was found that the An14g04280 overexpression strain, in contrast to the parent control strain, has a preference for D-galacturonic acid over D-xylose as substrate. Overexpression of this transporter in *A. niger* resulted in a strong increase of D-galacturonic acid uptake and induction of the D-galacturonic acid reductase activity, suggesting a metabolite controlled regulation of the endogenous D-galacturonic acid catabolic pathway.

## Introduction

Global limits in food and energy availability are becoming a major concern. Arable land is needed for production of food and large waste streams come from processing of our largest food resources, like grain, maize, potato and rice. These waste streams are currently insufficiently exploited and mainly used for feed and energy production (Howard et al. [2003]). Alternatively, agricultural waste streams can be used as substrate for fermentative production of chemicals by microorganisms.

Historically, *Aspergillus* species are used for the production of food additives and platform chemicals such as citric acid (*A. niger*) and itaconic acid (*A. terreus*) (Willke and Vorlop [2001]). The filamentous fungus *Aspergillus niger* is additionally exploited for production of enzymes for food and feed applications, many of which have been rewarded the GRAS (Generally Recognized As Safe) status (Howard et al. [2003]; van Dijck et al. [2003]).

Grain, maize and rice waste stream material contains around 40% cellulose, 35% hemicellulose, 20% lignin and 5% pectin, while waste streams from other plants, such as sugar beet and potato, contain around 20% cellulose and hemicellulose, less than 1% lignin and 30% to 40% pectin (Ángel Siles López et al. [2010]; Howard et al. [2003]; Micard et al. [1996]). *A. niger* has a high capacity for degrading the hemicellulose, pectin and, to a lesser extend, cellulose fractions as the genome of *A. niger* encodes multiple gene variants of the enzymes required for the efficient degradation of these polysaccharides. (van den Brink and de Vries [2011]).

D-glucose and D-xylose, released from plant wall material, are carbon sources that will yield high energy upon being metabolized. The genome of *A. niger* is wired to specifically produce and secrete the enzymes involved in degradation of the cellulose and hemicellulose fractions to release D-glucose and D-xylose. These sugars are then taken up and metabolized, before energy is invested in the release of other carbon sources from polysaccharides. This preferential uptake is regulated by the interplay of activating and repressing transcription factors that respond to extracellular concentrations of inducers and sugars.

Expression of genes that code for enzymes involved in cellulose and hemicellulose degradation in *A. niger* is regulated by the transcription factor XlnR (Gielkens et al. [1999]). XlnR is activated by inducers derived from xylan, like D-xylose, though at a higher concentration this activation is attenuated by CreA (de Vries et al. [1999]; Ruijter et al. [1999]; van Peij et al. [1998]). Tight regulation of the specific genes encoding proteins involved in the release, uptake and metabolism of sugars gives the fungus the ability to utilize the available sugars in a physiologically most efficient way. However, circumventing this regulatory mechanism would potentially lead to simultaneous uptake of all sugar substrates and improved fermentation yields.

Genes encoding proteins involved in the degradation of pectin and the D-galacturonic acid metabolic pathway are found to be highly upregulated when *A. niger* is growing in the presence of D-galacturonic acid, in comparison to when the fungus is growing in the presence of D-glucose or D-fructose (Table 1). Three genes among the genes that are upregulated are coding for proteins with a strong similarity to transporters (Martens-Uzunova and Schaap [2008]; van der Veen et al. [2009]).

**Table 1:** **Data used for selection of putative D-galacturonic acid transporters (Martens-Uzunova and Schaap**[2008]**)**

**Locus tag**	**Functional annotation**	**Relative expression t = 4 h after induction on**		
		**galA**	**poly-galA**	**pectin**
An07g00780	Major facilitator superfamily strong similarity to monocarboxylate transporter	23.10	9.98	5.27
An14g04280	Major facilitator superfamily strong similarity to hexose transporter	26.53	22.74	27.95
An03g01620	Major facilitator superfamily strong similarity to hexose transporter	6.50	1.345	37.54

In this study we have functionally identified the D-galacturonic mono-acid sugar transporter by overexpression in *A. niger*. The effect of the increased uptake of D-galacturonic acid on the preferential uptake and metabolism of substrates has been assessed by mixed sugar fermentations and enzyme activity measurements.

## Materials and methods

### Strains

All *Aspergillus* strains used are descendants of *A. niger* N400 (CBS 120.49). *A niger* strain NW185 *cspA1, fwnA1, goxC17, prtF28*) was derived from NW131 and has been described by Ruijter et al. [1999]. The recipient strain in all transformation experiments NW186 (*ΔargB; pyrA6; prtF28; goxC17; cspA1*) is an *argB* and *pyrA* derivative of NW185.

### Vector construction

**Figure 1:**
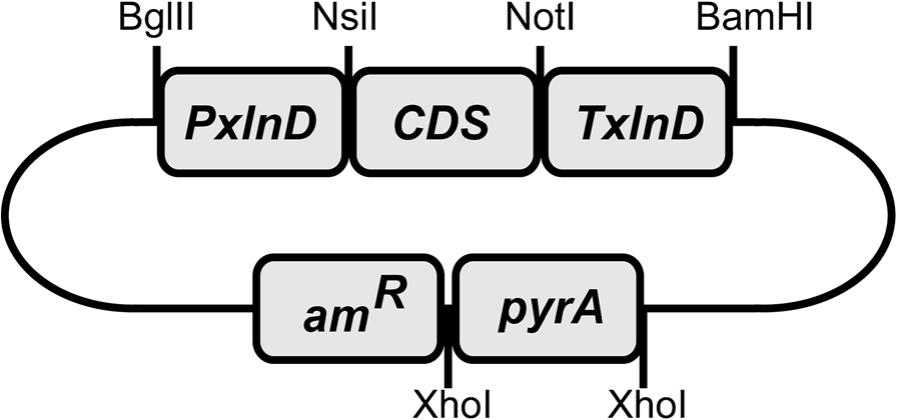
**Schematic representation of Funbrick expression vector.** Restriction sites flank functional modules. Capital P and T represent promoter and terminator. Bacterial marker: *amR*. Fungal uridine auxotrophy complementation marker: *pyrA*.

Primers used in the construction of vectors are summarized in Table 2. Plasmids used for transformations of *A. niger* were constructed according to the general scheme given in Figure 1. Martens-Uzunova and Schaap was the source for the selection of the putative transporters (Martens-Uzunova and Schaap [2008]); An14g04280 and An03g01620 were obtained from the *Aspergillus* genome database (Arnaud et al. [2012]; Pfaffl [2001]). These sequences were used to synthesize the respective coding sequences that were modified to remove conflicting restriction sites (BaseClear, Leiden, Netherlands). The An07g00780 fragment was amplified by PCR using primers JS_gatC_FW and JS_gatC_RV and *A. niger* NW186 genomic DNA as template and was subcloned into a pJET1.2 vector (Thermo scientific). The *BglII* restriction site was removed from the coding sequence by using the Quikchange Lightning Site-directed mutagenesis kit (Agilent Technologies) with the primers JS_gatCmut2_FW and JS_gatCmut2_RV. The PCR fragments were cloned into a pUC19 derived expression vector under control of an modified *xlnD* promoter and the *xlnD* terminator of *A. niger*. Plasmids were propagated in DH5α *E. coli*. The medium used for *E. coli* growth was LB (10 g/L Bacto tryptone, 5 g/L Yeast extract, 10 g/L NaCl) supplemented with 100 mg/L ampicillin when appropriate.

**Table 2:** Primers used in vector construction

**Primer**	**Sequence**
JS_*gatC*_FW	5’-GAGAATGCATATGTCTGAGCCTAAGAACCAGC-3’
JS_*gatC*_RV	5’-GAGAGCGGCCGCTCATATTTTGCTCGTATTCC-3’
JS_*gatC*mut2_FW	5’-GTTGCCAAAGTGGGAGACCTGCAAACCCTGGGTC-3’
JS_*gatC*mut2_RV	5’-GACCCAGGGTTTGCAGGTCTCCCACTTTGGCAAC-3’
JS_XlnDp_FW	5’-AGTCAATCGGTCATTCTCCG-3’
JS_*gatA*_RV	5’-CTCTTGGAGGACGCAAAACC-3’
JS_*gatB*_RV	5’-CATGGAAGCGGTGATCTAGG-3’

### Transformation of *Aspergillus niger*

For the transformation of *A. niger* NW186, protoplasts were generated using Novozyme-234 lysing enzyme cocktail. The An14g04280*,* An03g01620 and An07g00780 constructs were introduced in *A. niger* by transformation (Kusters-van Someren et al. [1991]) using the *pyrA* gene as a primary selection marker, relieving uridine auxotrophy. To generate the parent control reference strain, NW186 was transformed using pGW635, the resulting NW185 PYR A^+^ strain was used as a control strain in our studies. Colonies were randomly picked from the primary transformation plates and re-plated on selective medium to purify the single colonies.

### Analysis of *A. niger transformants*

Screening of randomly picked transformants was done by PCR on genomic DNA isolates. For that, fresh mycelium was disrupted using MP lysing matrix C tubes and 400 μl DNA extraction buffer (0.1 M Tris HCl pH 8.0, 1.2 M NaCl, 5 mM EDTA). DNA was extracted using phenol-chloroform extraction. The pellet was washed with 70% cold ethanol, air-dried and re-suspended in 50 μl MQ water. The presence of An14g04280*,* An03g01620 and An07g00780 expression constructs was confirmed by PCR using the JS_XlnDp_FW primer, binding to the promoter region of the construct, and JS_gatA_RV, JS_gatB_RV or JS_gatC_RV specifically binding to the complementary strand in the corresponding coding region. Transformants with confirmed gene constructs were re-plated on complete medium. Spores are harvested after 4 days of growth at 30°C.

### Growth in shake flasks

Strains were grown in duplicate in 200 mL PM medium (1.2 g NaNO_3_, 0.5 g KH_2_PO_4_, 0.2 g MgSO_4_^.^7 H_2_O, 0.5 g Yeast extract and 40 μg Vishniac per liter) (Ruijter et al. [1999]) with 91 mM sorbitol and 9 mM D-galacturonic acid or 100 mM of the synthetic cell wall hydrolysate (SCH) (Carpita and Gibeaut [1993]) (Table 3) as a carbon source in 1 L flasks. For inoculation, a final concentration of 1•10^6^ spores per mL was used. Induction of the *xlnD* promoter with 10 mM D-xylose was done at t = 0, 18 hours after inoculation (van der Veen et al. [2009]). Transformants were grown for 5 days at 30°C and 250 rpm. Medium samples were taken by filtration of 2 ml of the culture broth through a 5-micron pore size nylon filter. Biomass samples were obtained by washing of the retentate with demineralized water and flash freezing using liquid nitrogen. The samples were taken at T = −18 h, 0 h, 6 h, 30 h, 54 h for the first growth experiment on D-galacturonic acid. For the second D-galacturonic acid growth experiment, samples were taken at T = −18 h, each hour from T = 0 h to T = 10 h, 24 h, 30 h, 54 h and 78 h. For the growth experiment with SCH as a carbon source, samples were taken from T = 0 h with steps of 24 and 6 hours: T = 0 h, T = 24 h, T = 30 h, T = 48 h, T = 54 h.

**Table 3:** Composition of synthetic cell wall hydrolysate (SCH)

**Compound %**	**Concentration (g/l)**
D-glucose; 65%	13
D-xylose; 20%	4
D-galacturonic acid; 10%	2
L-arabinose; 3%	0.6
D-galactose; 1%	0.2
D-mannose; 1%	0.2

### Growth in fermentors

Strains were grown in duplicate in 500 mL PM medium (Ruijter et al. [1999]) with 100 mM sorbitol as a carbon source in 1 L flasks. For inoculation, a final concentration of 1•10^6^ spores per mL was used. Cultures were incubated for 18 hours at 30°C with 250 rpm orbital shaking. Mycelium was aseptically harvested by filtering over a sterile nylon cloth, washed with PM without carbon source and transferred to 1 L fermentors. Fermentors contained 750 ml PM with 50 mM D-xylose and 50 mM D-galacturonic acid, adjusted to pH6. Air was flushed at a stirrer speed of 600 rpm. Medium and biomass samples were taken as previously described, with an additional sampling of mycelium from 10 ml broth volume for enzymatic assays. Carbon dioxide and oxygen concentrations in off-gas were measured as well as pH.

### HPLC analysis

High-pressure liquid chromatography (HPLC) was used to determine the extracellular concentrations of D-glucose, L-sorbitol, D-xylose, citric acid and D-galacturonic acid in the culture broth samples. A Shodex KC-811 column was used at 30°C that was eluted with 0.01 N H_2_SO_4_ at a flow rate of 0.8 mL min^−1^. A refractive index detector (Spectrasystem RI-150, sample frequency 5.00032 Hz) and a UV–VIS detector (Spectrasystem UV1000, λ = 210 nm) were used for detection of the eluting compounds. Crotonate at a concentration of 6 mM was used as an internal standard.

### Enzyme assay

To determine the activity of the D-galacturonic acid metabolism, the first step in the enzymatic conversion, D-galacturonic acid to L-galactonic acid, was measured. D-galacturonic acid reductase assay based on conversion of NADPH to NADP^+^, previously described by Kuorelahti et al., was applied (Kuorelahti et al. [2005]). 20 mg of frozen mycelium was added to 400 μl of extraction buffer (100 mM phosphate buffer pH 7.0, 0.1 mM EDTA, 1 mM DTT and fungal protease inhibitor cocktail) in lysing matrix C tubes (MP biomedicals) and disrupted at level 6 for 30 seconds in a bead beater (MP fastprep-24). Cell debris was removed by centrifugation and supernatant was used as cell extract. 25 μl of cell extract was added to 200 μl of assay buffer (100 mM sodium phosphate buffer pH 7.0, 0.25 mM NADPH) and reaction was started by the addition of 25 μl of substrate (1 M D-galacturonic acid pH 6). Decrease of absorbance at 340 nm was measured on a platereader (Biotek Synergy) in parallel with sample controls, for which 25 μl of buffer was added. Background activity of sample control without D-galacturonic acid was substracted from sample measurements with D-galacturonic acid. Protein content was determined by photometric assay (Peterson [1977]).

## Results

### Strains that overexpress GatA have increased D-galacturonic acid uptake in shakeflask cultures

To study the effect of the expression of the putative D-galacturonic acid transporter encoding gene, the D-galacturonic acid concentration in the medium was measured during growth. A faster uptake of D-galacturonic acid from the medium was observed for the cultures with the An14g04280 overexpression strains (transformant) in comparison to the NW185 PYR A^+^ control strain. At 6 hours after induction, the strains that overexpress An14g04280 had taken up over 50% of D-galacturonic acid present at the start of the cultivation, while no D-galacturonic acid had been taken up by the control strain. The strains containing the other constructs did not show any difference in D-galacturonic acid uptake in comparison to the NW185 PYR A^+^ control strain. The effect of the An14g04280 overexpression strain is most pronounced between time points 6 and 30 h.

**Figure 2:**
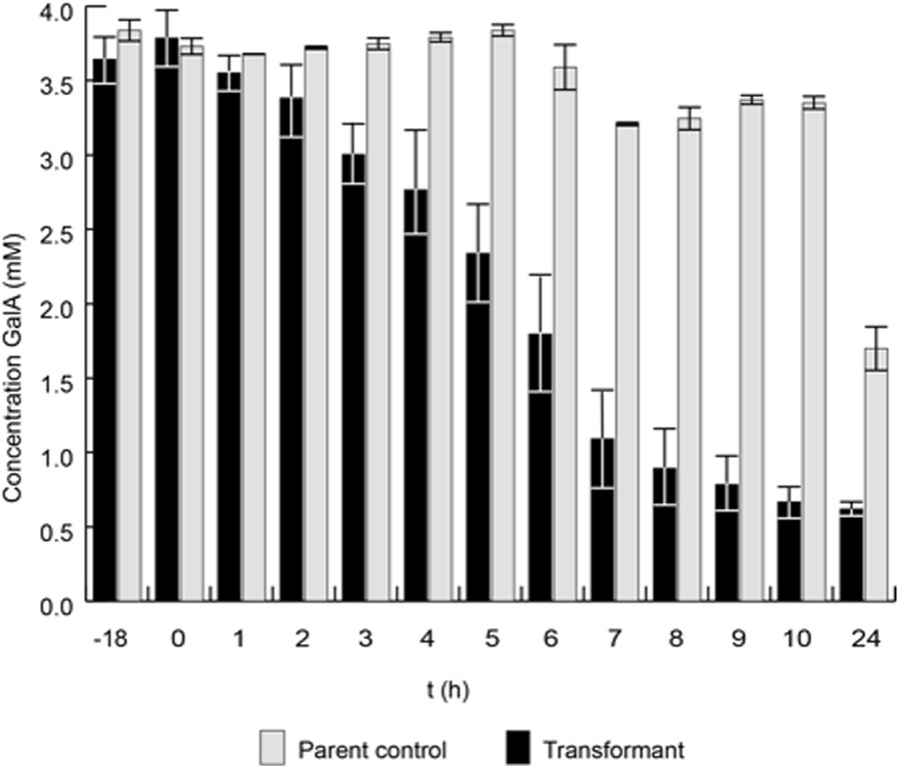
**Concentration of GalA in the medium during growth on sorbitol and GalA.** Induction of expression system with D-xylose at T = 0. Overexpression strains of *gatA* (number of replicates =4) in comparison to the control NW185 PYR A^+^ (number of replicates =2).

A second experiment was performed to get a more time resolved determination of the uptake of D-galacturonic acid by the best performing transformant strain (JS013). The difference in D-galacturonic acid uptake starts to become evident from 4 hours after induction, with the highest contrast between 5 and 6 hours after induction (Figure 2).

**Figure 3:**
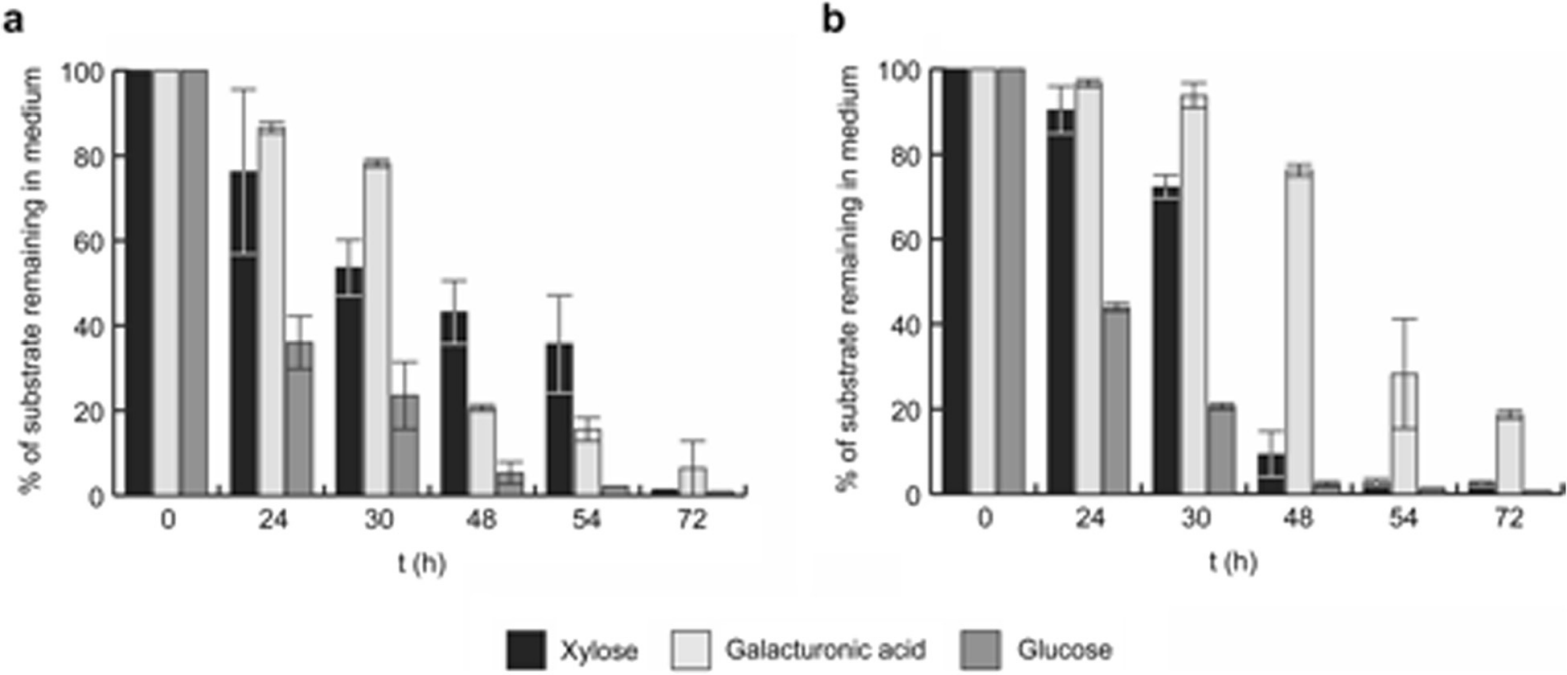
**Percentage remaining in medium of the three most important substrates from the synthetic cell-wall hydrolysate for** ***gatA*** **overexpression strain (a) (number of replicates =2) and the parent (NW185 PYR A**^**+**^**strain** ***)*** **(b) (number of replicates = 2).**

To study the effect of An14g04280 in the uptake of D-galacturonic acid during growth on more complex mixed sugar substrates, JS013 was grown in the presence of a substrate mix (SCH), mimicking the composition of a plant cell wall hydrolysate (Carpita and Gibeaut [1993]). When comparing the uptake of the three main components D-glucose, D-xylose and D-galacturonic acid between the transformants and the control strain, a clear difference is found (Figure 3a and b). Till 24 hours after inoculation, substrate uptake is similar for all strains. After 30 hours, differences in D-galacturonic acid and D-xylose uptake become evident. At time point 48 hours, the control strain has almost depleted the D-xylose and most of the D-galacturonic acid remains in the medium, while the transformant has consumed 80% of the D-galacturonic acid and most D-xylose remains in the medium. While D-glucose and D-xylose are depleted in the control culture after 54 hours, depletion of D-xylose in the transformant cultures can only be seen at time point 72 h.

**Figure 4:**
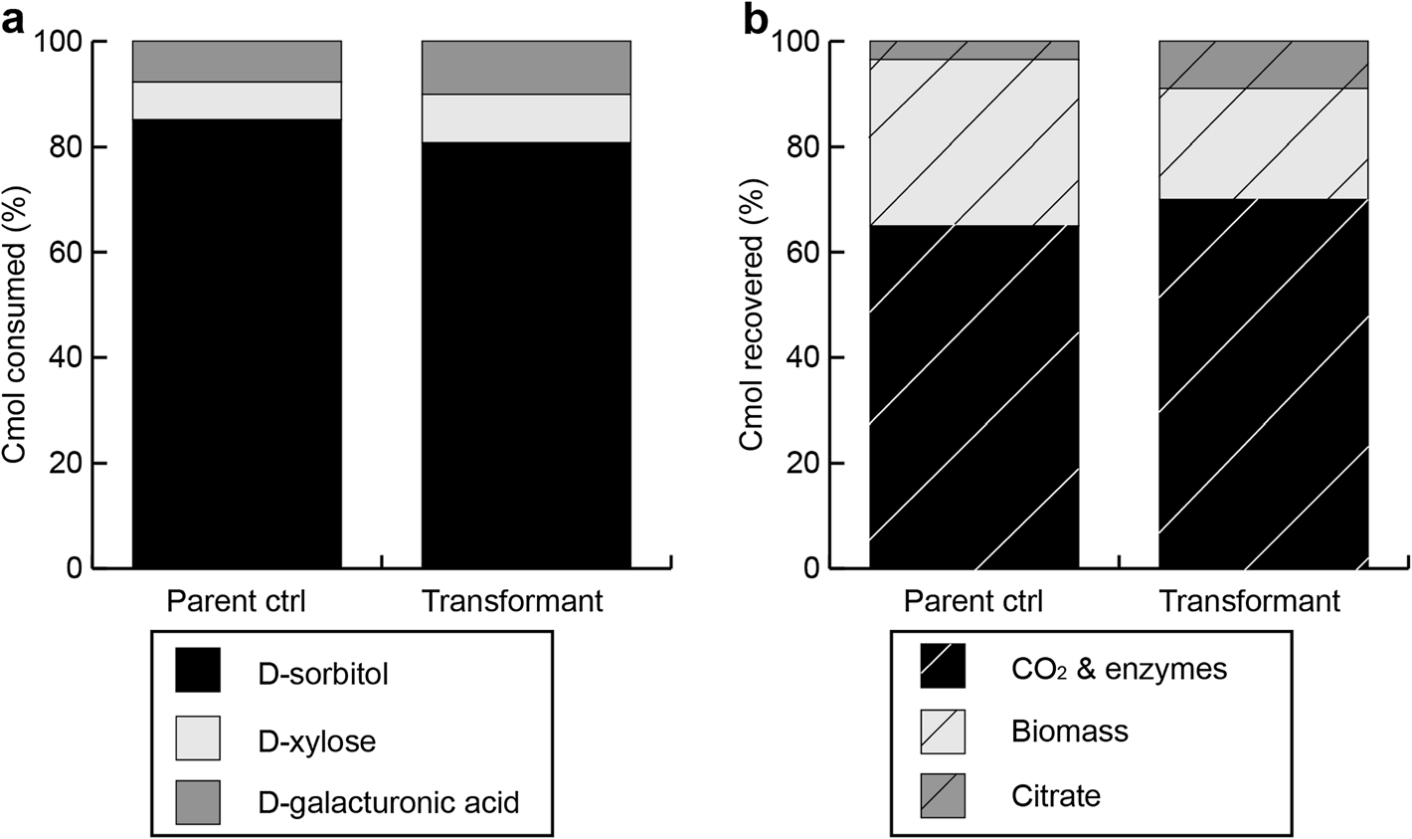
**Carbon consumed (a) and produced (b), expressed in Cmoles, for control strain (NW185 PYR A**^**+**^**strain) and transformant JS013 after 72 h of growth on SCH medium.** Samples taken from direct cultures, D-xylose in the mixed substrate medium serves as an inducer of the *xlnD* promoter. Totals represent 0.1 Cmoles.

The biomass formed and the citrate produced were measured for the An14g04280 overexpression strain and the control strain. Recovery of substrate in biomass and citrate produced was 35% for the control strain and 30% for the transformants. It was found that for the overexpression strain 30% of this is citrate and 70% is biomass, while for the control strain, 10% is citrate and 90% is biomass (Figure 4). Cmoles that are not accounted for are assumed to be CO_2_ and enzyme production (not measured). For these calculations the generalized molecular formula for fungal biomass CH_1.72_O_0.55_ N_0.17_ was used (Carlsen and Nielsen [2001]).

### Growth of transporter overexpression strains in fermentors

**Figure 5:**
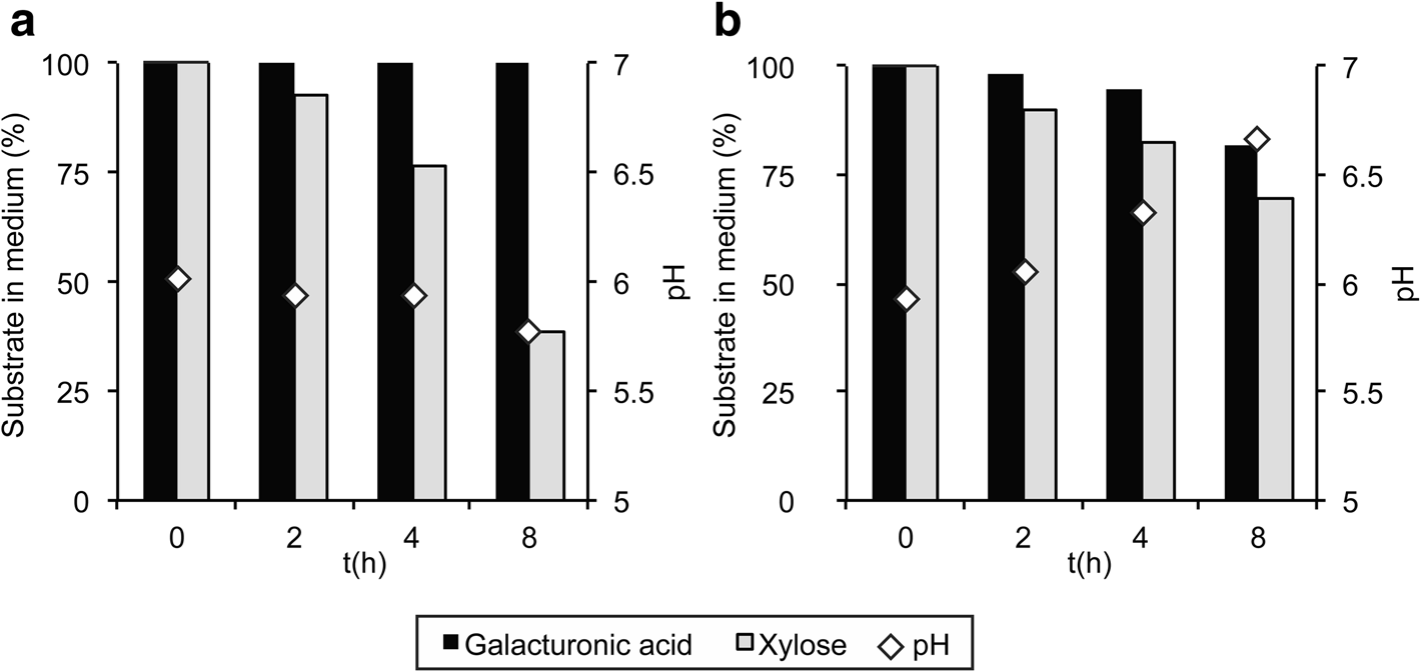
**Substrate remaining in culture medium and pH during growth in fermentors of the control, (NW185 PYR A**^**+**^**strain) (a) and overexpression strain, JS013 (b).** The strains were pre-grown and mycelium was transferred at T = 0 to fermentors containing D-xylose and D-galacturonic acid as substrates.

**Figure 6:**
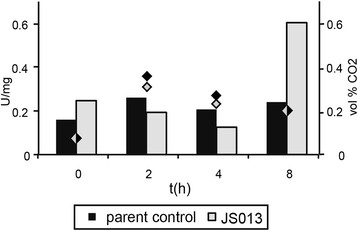
**Galacturonic acid reductase activity (Bars; left Y axis) and CO**_**2**_** in offgas (Diamonds; right Y axis).** Samples were taken during growth on 25mM of D-xylose and 25mM of D-galacturonic acid as a carbon source in fermentors.

When strains were grown in the presence of equimolar concentration of D-xylose and D-galacturonic acid in fermentors, preferential uptake of D-galacturonic acid over D-xylose of JS013 in comparison to NW185 PYR A^+^ control strain is observed. This is deduced from measured concentrations of D-galacturonic acid and D-xylose, but also from an increase of the pH of the medium, after D-galacturonic acid is taken up (Figure 5). Increased activity of the D-galacturonic acid metabolic pathway is observed for JS013 transformant strain in comparison to the control. For the first 4 hours after transfer to the fermentors, no difference in D-galacturonic acid reductase activity could be seen. 8 hours after transfer, however, specific activity of JS013 is two-fold higher than that of the control strain. The concentration of CO_2_ in the offgas went up to 0.35 percent by volume in the first two hours and slowly down to 0.2 percent by volume in the next 6 hours, while ambient concentration was 0.04 percent by volume (Figure 6).

### Phylogenetic analysis of GatA

**Figure 7:**
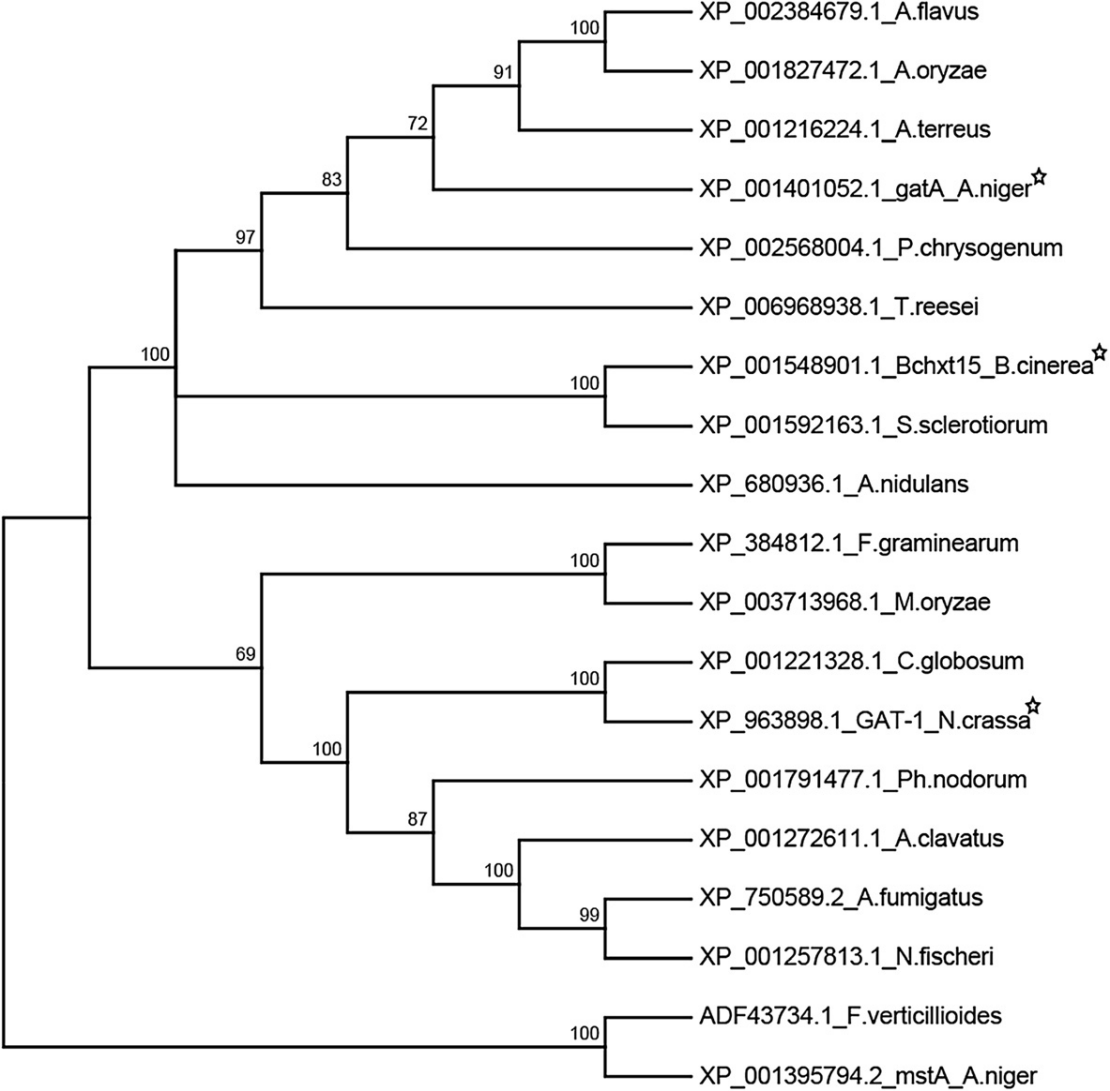
**Neighbor-joining tree of GatA and 17 putative orthologs, constructed with 1000 bootstrap replicates using MEGA6.06 (Tamura et al.**[2013]**), and a characterized high-affinity glucose transporter protein (*mstA_A.niger)*** **as an outgroup (vanKuyk et al.**[2004]**).** Stars indicate the three currently characterized D-galacturonic acid transporters. Bootstrap values are indicated at the branch-points. Labels indicate Protein Accession numbers followed by species abbreviations. *Abbreviations:Aspergillus clavatus; A. clavatus, Aspergillus flavus; A. flavus, Aspergillus fumigatus; A. fumigatus, Aspergillus nidulans; A. nidulans, Aspergillus niger; A. niger, Aspergillus oryzae; A. oryzae, Aspergillus terreus; A. terreus, Botrytis cinerea; B. cinerea, Chaetomium globosum; C. globosum, Fusarium verticilliodes; F. verticilliodes, Neosartorya fischeri; N. fischeri, Neurospora crassa; N. crassa, Penicillium chrysogenum; P. chrysogenum, Phaeosphaeria nodorum; Ph. nodorum, Sclerotinia sclerotiorum; S. sclerotiorum, Trichoderma reesei; T. reesei.*

**Figure 8:**
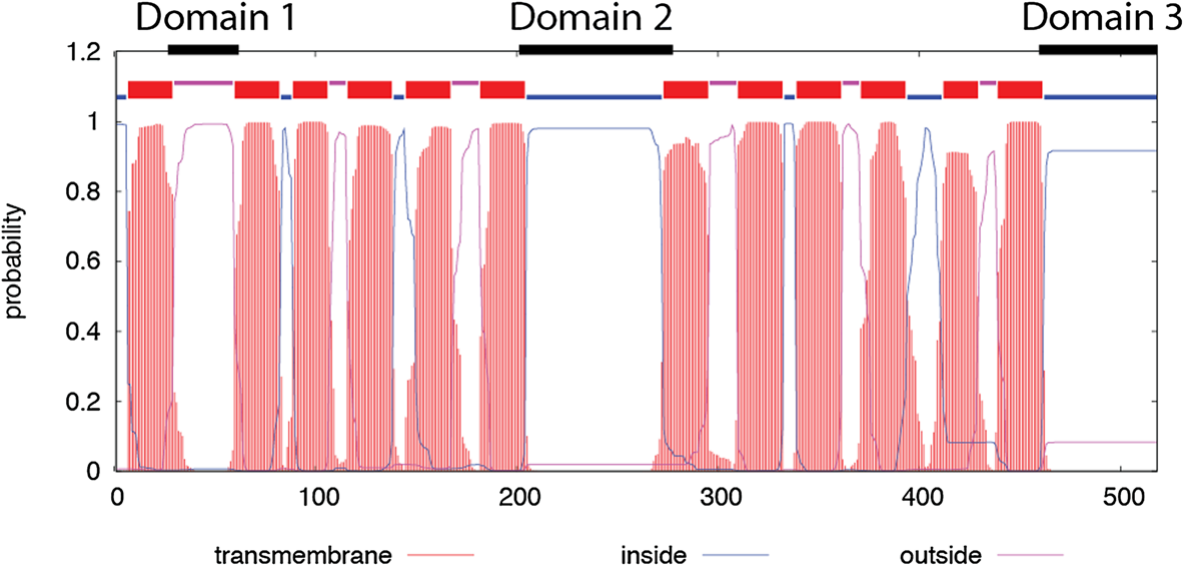
**Transmembrane domain prediction and indication of the two interacting domains (1, 3) and the putative substrate binding domain (2) used for comparison of the putative GatA orthologs (Viklund and Elofsson**[2004]**).**

In *Botrytis cinerea* and *Neurospora crassa*, D-galacturonic acid transporter proteins have been functionally identified (Benz et al. [2014]; Zhang et al. [2013]). After import, D-galacturonic acid is catabolized by three key enzymes: D-galacturonic acid reductase, L-galactonate dehydratase and 2-keto-3-deoxy-galactonate aldolase, in *A. niger* encoded by *gaaA*, *gaaB* and *gaaC* respectively. Orthologs of the *gaaA* and *gaaC* genes have been identified in *B. cinerea* and *N. crasssa* and 16 other fungal species that all have a conserved bidirectional organization of their *gaaA – gaaC* promoter region (Martens-Uzunova and Schaap [2008]). For each of these 18 species a bidirectional best BLAST hits procedure (Overbeek et al. [1999]) yielded a putative GatA ortholog. Orthologous amino acid sequences were aligned with Clustal (Larkin et al. [2007]) and a phylogenetic tree was constructed (Figure 7). All orthologs have 12 predicted transmembrane helices (Viklund and Elofsson [2004]) separating two interacting domains and the substrate binding domain (Yan [2013]) (Figure 8). In these three domains the three characterized D-galacturonic acid transporters share 18 conserved residues not present in the *A. niger* MstA D-glucose transporter protein sequence, which was used as an outgroup. The presence of this motif was used to assess the other 16 putative orthologs. Fifteen putative orthologs shared 8 of these residues, excluding the *Fusarium verticilliodes* putative ortholog which clusters with the D-glucose transporter MstA (Figure 7) and can be a transporter for another sugar.

## Discussion

Three genes that potentially encode the D-galacturonic acid transporter in *A. niger* are investigated. *A. niger* transformants that overexpress the An14g04280 gene under control of the D-xylose induced *xlnD* promoter show a significant increase in D-galacturonic acid uptake. From this we conclude that this gene, *gatA*, encodes for the D-galacturonic acid transporter, GatA.

By analysis of the amino acid sequence using the α-helical transmembrane protein topology prediction software (PRODIV-TNHMM) (Viklund and Elofsson [2004]) GatA is predicted to be part of the Major Facilitator Superfamily (MFS). MFS transporter proteins consist of 12 transmembrane helices and have both C- and N-termini located in the cytoplasm.

This study shows that the overexpression of *gatA*, using a D-xylose inducible promoter, leads to differences in the uptake of sugars when the strains are grown on various mixed sugar substrates. GatA overproducing strains preferentially use D-galacturonic acid over D-xylose, while the wild type strains prefer D-xylose. D-xylose is used for overexpression of the GatA transporter in all growth experiments and it is also used as a substrate. However, the main carbon source present in the medium is sorbitol, a non-inducing, non-repressing carbon source. Our extensive previous studies have shown that in this condition induction of expression occurs at concentrations of 0.1 to 50 mM D-xylose (van der Veen et al. [2009]; Mach-Aigner et al. [2012]).

The increased D-galacturonic acid uptake of the transformant was not reflected in the basal GaaA enzyme activity during the first hours of growth on D-xylose and D-galacturonic acid. It is, however, reflected in an increased GaaA enzyme activity after 8 hours. This time delay suggests that GaaA enzyme activity is not limiting D-galacturonic acid uptake and metabolism during the first few hours of growth on these substrates and that regulation of expression of GaaA is taking place “at the gate”, *via* import of D-galacturonic acid. The repression of induction of GaaA in the presence of D-xylose in the parent strain, is bypassed by an increased influx of substrate in the GatA overexpressing strain, suggesting that the endogenous D-galacturonic acid catabolic pathway is controlled by a pathway intermediate such as L-galactonate or keto-deoxy-L-galactonate as has been suggested for *T. reesei* (Kuivanen et al. [2012]).

Several repressing and inducing regulatory systems are known to be functioning in *Aspergillus niger*. The glucose carbon catabolite repressor *creA* is the most studied and is known to be dominant in most cases (de Vries et al. [1999]). The promoter region of the *gatA* gene shows two consensus sequences for binding of this protein, which suggests that D-glucose is repressing the expression of the endogenous gene. The *xlnR* xylanolytic induction system is also well studied and an inducer consensus binding sequence is known (van Peij et al. [1998]). This consensus sequence can not be found in the upstream region of the endogenous *gatA* gene and it is therefore not induced by the presence of D-xylose. Additionally, GatA expression has been found to be stricktly coregulated with D-galacturonic acid catabolic enzymes and a number of extracellular galacturonases (Martens-Uzunova and Schaap [2008]). The regulator of the D-galacturonic acid metabolism, however, remains unknown for now.

We have investigated the application of our findings by growing our strains on a synthetic medium mimicking a plant cell wall hydrolysate. Under these conditions the *gatA* overexpressing strains preferentially use D-galacturonic acid over D-xylose. In these experiments it was found that GatA overexpression strains had a higher citric acid yield. While the effect seems pronounced on a simulated hydrolysate substrate, further experiments are needed to study the benefit on second generation feedstocks with high D-galacturonic acid content like sugar beet pulp to investigate the potential of GatA in biotechnological applications.

## Competing interests

The authors declare that they have no competing interests.

## Authors’ contributions

JS and LG designed the study. JS and MS designed and performed the experimental work. PS contributed to the design and execution of the phylogenetic analysis. JS wrote the manuscript PS and LG participated herein. All authors read and approved the submission of the manuscript.
